# Comparison of Skin Lesions Caused by *Ixodes ricinus* Ticks and *Lipoptena cervi* Deer Keds Infesting Humans in the Natural Environment

**DOI:** 10.3390/ijerph17093316

**Published:** 2020-05-10

**Authors:** Weronika Buczek, Alicja M. Buczek, Katarzyna Bartosik, Alicja Buczek

**Affiliations:** Chair and Department of Biology and Parasitology, Medical University of Lublin, 20-080 Lublin, Poland; wera1301@gmail.com (W.B.); abuczek21@gmail.com (A.M.B.); katarzyna.bartosik@umlub.pl (K.B.)

**Keywords:** castor bean tick, deer ked, *Ixodes ricinus*, *Lipoptena cervi*, tick bite, deer ked dermatitis

## Abstract

*Background*: The territorial expansion and increased population size of haematophagous arthropods (i.e., the castor bean tick *Ixodes ricinus* (Ixodida: Ixodidae) and the deer ked *Lipoptena cervi* (Diptera: Hippoboscidae)) has enhanced the risk of human infestations in Europe. The aim of our study was to present skin lesions induced by tick and deer ked bites in patients from recreational forest regions in southeastern Poland and pay attention to features of skin changes that may be useful in differential diagnosis. *Methods*: We compare the skin lesions after *I. ricinus* and *L. cervi* bite and draw attention to the biological and ecological traits of both ectoparasites, which may be diagnostically relevant for determination of the cause of skin symptoms reported by patients. *Results*: *I. ricinus* bites lead to development of erythematous-infiltrative poorly demarcated lesions with a centrally located bite mark, which usually disappears within one to several days. In turn, *L. cervi* bites leave irregularly shaped scattered erythematous papules. The papules may persist for up to one year and are accompanied by itching. *Conclusions*: Correct assessment of the clinical picture and its association with an arthropod bite (e.g., tick or deer ked) is highly important for further diagnostic procedures (i.e., differentiation of skin lesions developing in tick-borne diseases and, consequently, correct choice of pharmacological therapy). *I. ricinus* and *L. cervi* differ in their developmental cycles and rhythms of activity, which indicates that both species should be considered potential causative agents in the differential diagnosis of skin lesions when the patient has been bitten by an arthropod in autumn and winter months.

## 1. Introduction

Humans can be attacked by a variety of haematophagous arthropods (e.g., ticks from the genus *Ixodes* (Ixodida: Ixodidae) and *Lipoptena* deer keds (Diptera: Hippoboscidae)) which exist on many continents, mainly in temperate climate zones. Two representatives of these two genera (i.e., the castor bean tick (*Ixodes ricinus*) and the deer ked (*Lipoptena cervi*)) are the most widespread species in Europe. Their occurrence in various habitats is associated with the presence of potential animal hosts, whose ranges have expanded dynamically due to changes in the environment caused by anthropogenic factors. The large group of *I. ricinus* hosts comprises many species of terrestrial vertebrate animals. Additionally, all developmental stages of the tick (i.e., larvae, nymphs, and adults), obligatorily ingest the blood of different animals. In the case of *L. cervi*, only female and male adults are ectoparasites and collect blood from cervids, mainly the roe deer (*Capreolus capreolus*) and the moose (*Alces alces*). The hair coat of these hosts, where females deposit one puparium, is the site of reproduction of this dipteran species [1]. These parasites can also feed on other animals such as cattle (*Bos taurus*), sheep (*Ovis aries*), and horses (*Equus caballus*), or even on companion animals, such as dogs. With their similar ecological requirements and host preferences, active stages of *I. ricinus* and *L. cervi* colonize the same habitats, which implies that humans can be attacked by both species in the same area.

The extension of the distribution ranges and the growing numbers of *I. ricinus* ticks and deer ked in Europe increases the risk of human infestations by these arthropods [2,3]. In the last two decades, the number of patients suffering from tick-borne diseases transmitted by *I. ricinus* (i.e., borreliosis, rickettsiosis, and tick-borne encephalitis (TBE)) has increased significantly. The role of *L. cervi* in the transmission of pathogens is still poorly explored. Most research has been focused on the potential transmission of *Bartonella* bacteria by these blood sucking insects and the role of their hosts in the maintenance of these pathogens in nature [4,5,6,7,8,9,10,11,12]. DNA of several human pathogens—most often *Bartonella schoenbuchensis* as well as *Anaplasma phagocytophilum*, *Rickettsia* spp., *Coxiella* spp., and *Borrelia burgdorferi*—have been detected in *Lipoptena* species (e.g., [13,14,15,16,17,18]).

The direct effects of the parasitism of these arthropods on humans and animals include local and/or systemic reactions induced by the components of their saliva introduced during blood ingestion.

Although increasing numbers of attacks on humans by *I. ricinus* ticks and *L. cervi* are being continuously reported, the literature provides few descriptions of skin lesions caused by both species. A description of the picture of skin lesions caused by various species of haematophagous arthropods may be useful for the differential diagnosis of human dermatitis.

To address this issue, the present study describes characteristic skin symptoms induced by tick and deer ked bites. Based on the results of our investigation and literature data, we indicate the biological and ecological traits of both arthropods. The knowledge of these characteristics can help determine the cause of skin lesions reported by patients and protect humans against arthropod attacks.

## 2. Materials and Methods

### 2.1. Study Area

The analyzed skin lesions were observed in patients staying in an area where *I. ricinus* and *L. cervi* coexist in Lublin Province (southeastern Poland) during the period of seasonal activity of these species. The area has large complexes of pine forests as well as local alder, oak-hornbeam, and ash-alder riparian forests. The fauna comprises large populations of cervids, including the roe deer (*Capreolus capreolus*), the red deer (*Cervus elaphus*), and the moose (*Alces alces*). These are all potential hosts of *L. cervi* adults and *I. ricinus* adults and nymphs, and many species of medium-size and small mammals such as hosts of juvenile tick stages. These habitats are often visited by local residents, mushroom and forest fruit collectors, and forest workers.

### 2.2. Clinical Cases

The first patient, a 56-year-old man, was bitten by a tick in May 2018 in the forest complex of Polesie Lubelskie (51°42′ N, 23°20′ E). The tick was removed with tweezers and was identified based on its morphological features as an *I. ricinus* female. The length of attachment of the female tick in the patient’s skin was estimated based on the epidemiological history including the date of the patient’s presence in the tick habitat and on the morphometric features of the removed specimen, which had been previously specified for the different phases of *I. ricinus* feeding [19]. The female was partially engorged and, according to our classification, it was removed in the second feeding phase. The size of *I. ricinus* females in the second feeding phase increases significantly compared to the size of females during the first two days after attachment, and their weight varies from 0.0017 to 0.3075 g (mean 0.0263 g). No infection as a result of the transmission of tick-borne pathogen was confirmed.

The other patient, a 15-year-old woman, was bitten by a deer ked at the beginning of November 2019 near Polesie National Park (51°24′ N, 23°12′ E). After short blood ingestion, the deer ked detached from the skin, and it was not possible to subject the specimen to molecular studies. Before the incident was reported, the patient had not used any medication to relieve the symptoms.

## 3. Results and Discussion

Visitors to forest and suburban recreational areas are at risk of being attacked by dipteran parasites *L. cervi* and *I. ricinus* ticks. Forest workers, hunters, farmers, and animal breeders often stay in arthropod habitats or have contact with animals infested by these parasites, which makes them a high-risk group. Based on the two patient cases and literature data, this short communication compares clinical symptoms in humans bitten by a deer ked and a castor bean tick.

**Figure 1:**
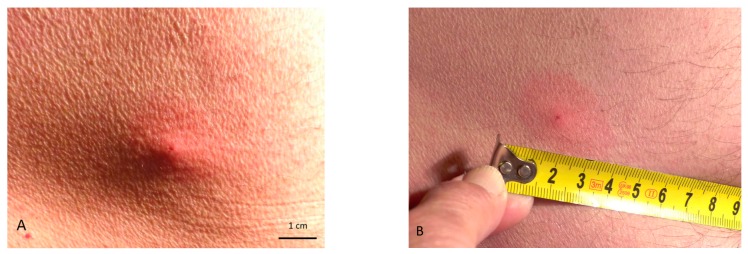
(**A**) A site of an *Ixodes ricinus* tick bite in the central part of the erythematous-infiltrative lesion, not sharply demarcated from the surrounding normal skin just after tick removal. (**B**) Lesion erythematous-infiltrative lesion with a diameter of 3 cm clearly demarcated from the surrounding normal skin. In the central part the infiltration decreased 48 h after tick removal.

An erythematous-infiltrative non-demarcated lesion with a diameter of approximately 3 cm appeared near the site of attachment of the female *I. ricinus* tick. The tick feeding site was visible in its central part (Figure 1). The skin lesion slightly changed within 48 h after removal of the tick but later faded gradually within three days. The patient did not feel pain or itching. Local reactions with predominance of erythema after *I. ricinus* bites are the most common symptoms in patients in southeastern Poland. As demonstrated by our previous investigations, they are reported by 57.6% of patients and by 20.1% of patients with additional systemic symptoms such as headache (10.8% of patients), fever (5.4%), lymph node enlargement (5.9%), or arthralgia (4.3%) [20].

**Figure 2:**
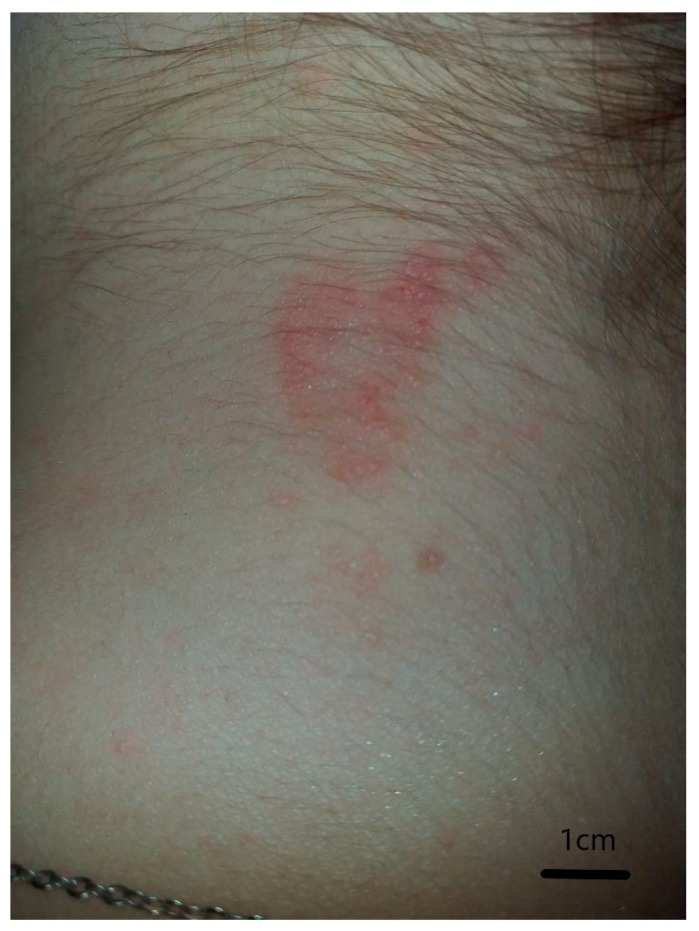
Scattered papules on an erythematous, irregular base after feeding of deer ked *Lipoptena cervi*, four weeks after the bite.

After the deer ked attack, erythematous papules that were irregularly shaped and scattered were observed on the patient’s skin (Figure 2). They persisted for at least eight weeks and were itchy. As demonstrated by other studies, papules formed from deer ked bites can persist for up to a year [4].

The skin lesion of deer-ked bites is probably of hypersensitivity origin [21,22]. Dermatitis in humans caused by *L. cervi* was most commonly described in Scandinavia [22,23,24]. Rantanen et al. [22] have observed intense pruritus, leading to scratching, erosions, and secondary infection by staphylococcal bacteria in many patients in Finland. Dermatitis which occurs after the bites, can range anywhere from a few to 50 red papules. These can range in size from a few mm up to 2 cm [23]. In this study, we described such a case for the first time in Poland.

As shown by our interviews with hunters who have been repeatedly attacked by deer keds, skin lesion may not appear after subsequent infestations at all, which may indicate development of resistance to the components of the saliva of this dipteran parasite.

The differences in the picture of skin lesions caused by *Ixodes* and *Lipoptena* infestations (Table 1) are probably related to the composition and amount of secreted saliva and the different physiology of feeding. *I. ricinus* females feed for 8–10 days [25]. During this period, they alternately introduce a portion of saliva and collect a portion of blood from hemorrhage sites. The composition and dynamics of secretion of saliva by tick salivary glands changes with the length of foraging. Unlike ixodid ticks, adult *Lipoptena* forms feed for a short period from 15 to 25 min, ingesting an amount of blood required for the reproduction and development of larvae [1]. The mouthparts of the deer ked called the haustellum and the elements of tick mouthparts (i.e., teeth-equipped chelicerae and the hypostome), are adapted to cut and attach to the skin throughout the feeding period. Both arthropods ingest host’s blood released from damaged blood vessels rather than directly from a blood vessel as in the case of mosquitoes. There are other factors that determine the picture and size of skin lesions in humans as well, such as the intensity of the invasion, the site of attachment of the arthropod on the host’s body, and the individual characteristics of the host such as immune status. 

Identification of the causative factor of the skin lesion is important for further diagnostic and therapeutic procedures. Due to the high vector competence of ticks, it is advisable to carry out serological tests after infestation of the patient’s skin to detect the presence of antibodies against pathogen antigens that may be introduced together with saliva during feeding. Tick or deer ked dermatitis should also be differentiated from skin lesions developing in the course of various tick-borne diseases (e.g., borreliosis (60%–80% of patients), rickettsioses (70%–80% of patients), ehrlichiosis (<30% of adults and <60% in children), and anaplasmosis (<10% of patients)) [26].

One of the most common tick-borne diseases worldwide is borreliosis (or Lyme disease) caused by *Borrelia burgdorferi* sensu lato, with erythema migrans (EM) as an early symptom. In contrast to skin lesions in humans developing in response to the active ingredients of saliva immediately after infestation by deer keds or ticks, EM with a diameter of over 5 cm (2 to 2.5 inches) appears between 3 and 30 days (most often after 7–10 days) after the tick bite and gradually increases to over 30 cm or more (12 inches or more). EM can have many forms [27]. The best known is a roughly circular red rash, usually with a lighter spot in the center (bull’s eye rash) without itching or pain. The central clearing in the borreliosis lesion is more common in patients in Europe (it occurs on average in 79% of cases) and non-endemic areas of the United States (on average 80%) than in the endemic areas of the United States (on average in 19% of cases) [28]. EM may also develop as a red, expanding lesion with central crust, multiple red lesions with dusky centers, or a bluish hue without central clearing [29]. Skin manifestations of Lyme disease also include multiple erythema migrans lesions (early disseminated stage), borrelial lymphocytoma, and acrodermatitis chronica atrophicans (late disseminated stage) [30]. A valuable clue can be provided to the doctor and diagnostician when the removed specimen is brought in for species identification. Due to the short feeding time of deer keds, it is usually not possible to collect specimens for diagnostic purposes.

Most frequently, ticks attach to the lower extremities, chest, and upper extremities [20,31], while deer keds attack the upper body parts, more often the back side of the body than the front side [32]. We observed attachment of deer keds to exposed areas of the human body, mainly on the back of the head and neck.

**Figure 3:**
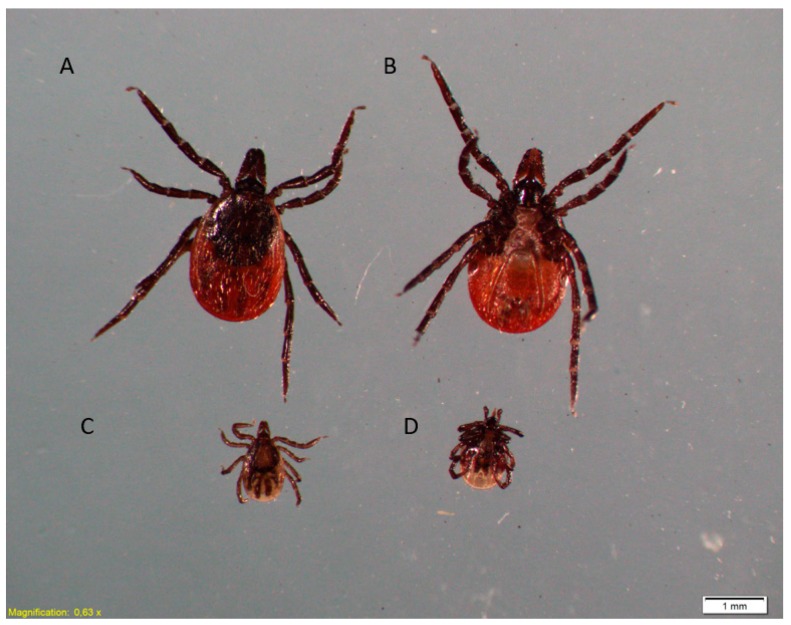
*Ixodes ricinus* female. Dorsal side (**A**) and ventral side (**B**). Nymph. Dorsal side (**C**) and ventral side (**D**).

**Figure 4:**
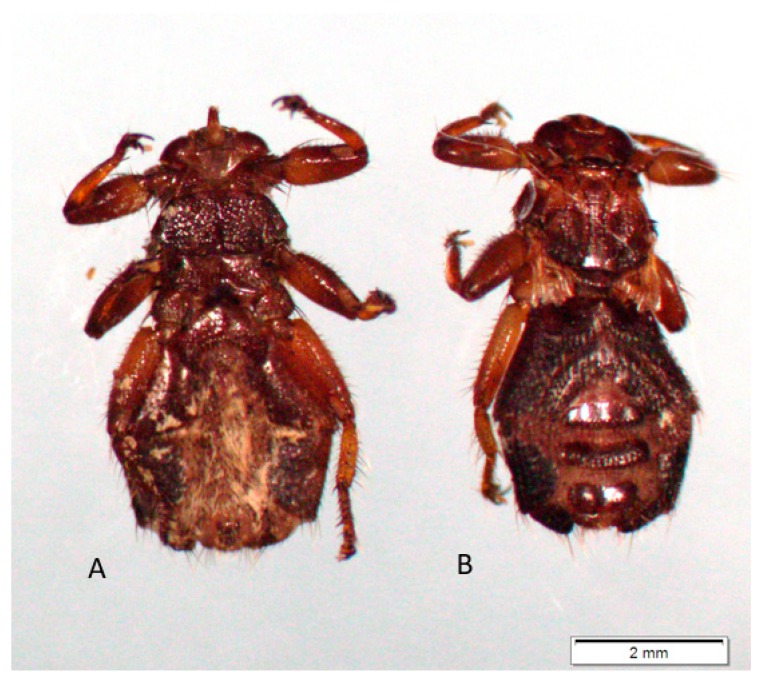
Female *Lipoptena cervi*. Ventral side (**A**) and dorsal side (**B**).

The number of patients presenting with skin lesions after tick and deer ked bites changes seasonally, with the highest values noted in periods of their increased presence in habitats during peak activity of these arthropods. The rhythms of questing activity are determined by the arthropod’s development cycle and climatic conditions. In Poland, *I. ricinus* nymphs and females (Figure 3), which infest humans most often, are active from April to September, with two peaks in May and September or one peak in May. In turn, parasitic winged *L. cervi* adults emerge from the pupa in September–December and then quest for a source of food (Figure 4). The diagnosis of skin lesions caused by arthropods still poses difficulties. Due to some similarities of some symptoms, skin lesions caused by deer keds may be confused with lesions caused by bites of other haematophagic arthropods, such as fleas. In both cases, patients develop skin itching. However, fleabites induce development of small red round-shaped bumps with a red “halo” around the bite centre on the skin. Traces of fleabites are arranged linearly or in clusters of three or four. The location of skin lesions caused by fleas and deer keds varies. Fleas usually bite on legs and ankles and less often in the groin, waist, and chest and in the folds of elbows and knees. Allergic reactions after fleabites may persist up to 24 h.

In the diagnosis of skin lesions in patients, valuable information about their cause can be provided by epidemiological history, which helps to determine the circumstances of the arthropod bite (place and time of year of the arthropod attack, habitat type, date of arthropod bite) excluding or confirming the possibility of attack by a specific arthropod species.

Given the harmful effects of haematophagous arthropods (i.e., ticks and deer keds) on humans, prophylactic methods that contribute significantly to reduction of their attacks should be applied. This includes avoidance of habitats during periods of activity of these arthropods, mainly at the peak of their activity. It also means protecting against bites by wearing body protection clothing and inspecting the body and clothes after returning home. The risk of tick bites can also be reduced by using repellents.

## 4. Conclusions

The abundance of *I. ricinus* and *L. cervi* in the same areas in Eurasia suggests that these ectoparasites should be considered as potential causative agents of skin lesions in patients. After a tick bite, a non-itching erythematous-infiltrated non-demarcated lesion develops and usually disappears after one day or several days. In turn, papules that appear on the skin after a deer ked bite persist for a long period of several weeks to a year and are accompanied by itching. Diagnosing dermatitis should be based on the clinical picture of these lesions, patient’s history with information about the circumstances of arthropod attacks (mainly place and time of year), and identification of the arthropod species removed from patient’s skin.

## Figures and Tables

**Table 1:** Differences between skin lesions caused by bites of *Ixodes ricinus* and *Lipoptena cervi*.

	*Ixodes ricinus*	*Lipoptena cervi*
Most frequent localization on the human body	Lower extremities, chest, abdomen, and upper extremities in adult patients. In children, it’s the same as adults and additionally the head and neck as well as the genital area.	Upper body parts (most frequently the back of the head and neck).
Type of skin lesions	Clearly visible puncture; erythematous infiltrated non-demarcated lesions.	Invisible or poorly visible puncture; irregularly shaped scattered erythematous papules.
Symptoms of a bite and development of skin lesions	The bite is painless and non-itchy; after detachment of an engorged tick or mechanical removal, an itching, burning, or red spot may sometimes develop; inflammation usually resolves after a few days, but may last longer in sensitive individuals.	Pain and itching may appear immediately after the bite; skin lesions persist for a long time, from several weeks to a year.
Time of exposure to bites	Throughout the seasonal activity of nymphs and adult stages, mainly at its peaks (i.e., in May and September).	Period of activity of adult stages from September to December.
